# Double-Sided Suspending Evaporator with Top Water Supply for Concurrent Solar Evaporation and Salt Harvesting

**DOI:** 10.1021/acssuschemeng.2c03948

**Published:** 2022-09-13

**Authors:** Xiaolong Ma, Xiaodong Jia, Guice Yao, Dongsheng Wen

**Affiliations:** † School of Chemical and Process Engineering, University of Leeds, Leeds LS2 9JT, U.K.; ‡ School of Aeronautical Science and Engineering, Beihang University, Beijing 100191, China; § Lehrstuhl für Thermodynamik, Technical University of Munich, Garching 85748, Germany

**Keywords:** solar evaporation, salt harvesting, suspending evaporator, desalination, water distribution system

## Abstract

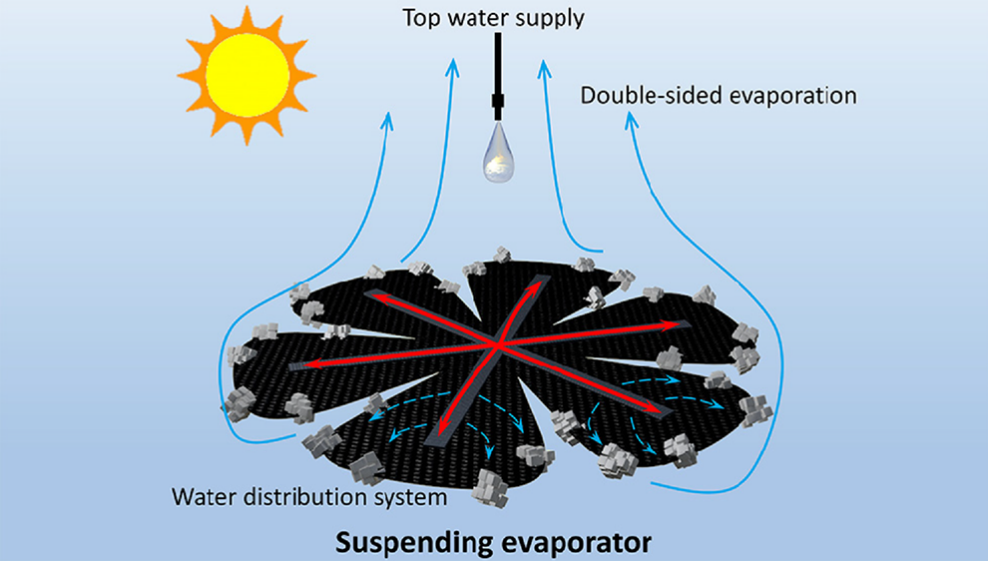

Solar evaporation of seawater is promising to mitigate the fresh water scarcity problem in a green and sustainable way. However, salt accumulation on the photothermal material prevents the system continuous operation, and the water supply driven by capillary force severely limits the scale-up of the evaporators. Here, we demonstrate a double-sided suspending evaporator with top water supply and a surface water distributor for high-efficient concurrent solar evaporation and salt harvesting for large area applications. Both sides of the evaporator can evaporate water with automatic salt harvesting from the edge concurrently. Top water supply gets away from the limitation of capillary force for a larger area application and completely cuts off the heat leak to the bulk water below for higher efficiency. The energy conversion efficiency reaches 95.7% at 1.40 kg·m^–2^·h^–1^ with deionized water under 1 sun with a remarkable low surface average temperature (28.2 °C). Based on the simulation and experiment, a novel radial arterial water distribution system is developed to efficiently distribute water on a larger evaporation surface. The water distribution system alters the water transport path in the evaporation surface, leading to salt accumulation on the surface body, where salt is unable to be harvested by gravity automatically. This problem is further resolved by cutting out the salt accumulation area (16.4%) on the surface to create a floriform evaporator, which forcedly exposes the salt at the edge for harvesting. Up to70 h continuous solar evaporation from salt water at a rate of 1.04 kg·m^–2^·h^–1^ with concurrent salt collection on this floriform evaporator is achieved. This work resolves water supply and salt accumulation problems in scaling up the solar evaporators and advances the structural design of evaporators for high-efficient large area applications.

## Abstract 2

A double-sided suspending evaporator with top water supply and water distributor is proposed for large area solar evaporation and salt harvesting.

## Introduction

Solar evaporation utilizes photothermal materials to convert solar energy into heat for water evaporation and generates high-quality drinkable fresh water.^1−3^ It is a promising desalination technology to mitigate the fresh water scarcity problem in a green and sustainable way.^4−7^ In this technology, photothermal materials have received extensive interest.^8−10^ These materials can be broadly classified into three categories: carbon-based materials, for example, carbon black, graphite, carbon nanotubes, graphene, graphene oxide, and reduced graphene oxide^11−16^ plasma-based materials, for example, metals, metal oxides, and metal nitrides;^17−22^ and polymer-based materials, for example, polypyrrole and polydopamine.^23−25^ However, the solar absorptance of the material used for the first generation of the interfacial evaporator (IE) in 2014 reached 97% by using exfoliated graphite,^26^ and common carbon black can also reach 97%,^27^ which indicates that there is little room left for the improvement in material absorptance to increase the evaporation system efficiency. The energy conversion efficiency for the evaporation system is determined by two major factors: solar absorptance of the photothermal materials (from solar energy to heat) and vapor generation efficiency of the evaporator (from heat to vapor). Compared to the intensive study of photothermal materials, the investigation of the structural improvement of evaporators is still insufficient. Proper structural optimization could improve the vapor generation efficiency by reducing heat loss, supplying sufficient water for evaporation and improving the vapor transport conditions.

A typical IE floats at the water–air interface, with a top solar absorbing layer, and a thermal insulation layer below to localize the heat on the evaporation surface.^28−30^ Water supply is typically driven by capillary force originating from the middle porous layer. One of the key design considerations of IE is to reduce heat leak into the bulk water, which relies on the thermal insulation layer. Either the waterproof insulation layer or the porous insulation layer has been used. Employing waterproof thermal insulation layers reduces heat leak but forces water to go through the side walls to the evaporation surface center,^31,32^ which can hardly satisfy the evaporation rate on a larger evaporation surface. Using a porous thermal insulation layer allows the water below to be directly transported to the evaporation surface, which solves the water supply issue for large evaporation surfaces,^13,33^ but greatly suffers the heat loss problem through water channels. More importantly, when applied on seawater, salt would accumulate on the photothermal materials and gradually stop the system.^34−37^

Recently, evaporators with central water supply are receiving great interest. It uses a central archive structure to supply water to the evaporation surface and accumulates salts at the edge, where salts can automatically fall down by gravity.^38−41^ Based on this central water supply design, an umbrella evaporator was further proposed, whose both sides of the evaporation surface contribute to evaporating without using thermal insulation layers, and salts accumulate at the edge and fall down after the dissolution of the connecting parts.^42^ The structure improvements lead to impressive results for continuous water evaporation and salt harvesting concurrently. However, their water supply, which is driven by capillary force, cannot keep up with a higher evaporation rate, which greatly limits the scale-up of the evaporators for large area applications.

To address the heat loss, salt accumulation, and scale-up problems, we developed a double-sided suspending evaporator with top water supply and surface water distribution system for high-efficient concurrent solar evaporation and salt harvesting for large area applications. The water supply for this evaporator is from the top and is dripped onto the evaporation surface center, which gets away from the capillary limitation while completely cuts off the heat loss to the bulk fluid below. Owing to the central top water supply, salt accumulates at the evaporation surface edge and falls down automatically due to gravity for harvesting. The evaporation rate reached 1.40 kg·m^–2^·h^–1^ with deionized water under 1 sun, whose corresponding energy conversion efficiency is 95.7%, with a remarkable low surface average temperature of 28.2 °C. Through both simulations and experiments, we further designed a radial arterial water distribution system to efficiently distribute the water on a larger evaporation surface. The water distribution system leads to salt accumulation on the surface body, where salt is unable to be harvested by gravity automatically. By cutting out the salt accumulation area (16.4%), we developed a floriform evaporator (11 cm in diameter) and achieved 70 h continuous solar evaporation and salt harvesting at 1.04 kg·m^–2^·h^–1^ with 3.5 wt % NaCl solution with it. This work advances new ideas for the structural design of the evaporators for high-efficient large area applications.

## Experimental Section

### Evaporation Experiment

Polypyrrole was coated on a filter paper to obtain a black evaporation surface (0.16 mm in thickness), whose solar absorptance is 96.2% and porosity is 0.806, which can be found in our previous work.^42^ The evaporator was supported by nylon strings, which do not absorb water. A syringe pump that can control flow rate was used to supply water to the evaporation surface under a solar simulator (1 kW/m^2^). The ambient temperature was around 21 °C and the humidity was around 50% during the test. The water supply rate should meet the following two requirements: no dry part on the top evaporation surface and no water accumulation on the back surface by observation. The water supply rate was determined by trial and error to find a stable rate for different geometries based on the two requirements.

### Energy Conversion Efficiency Calculation

The energy conversion efficiency was calculated by the following equation:^43^
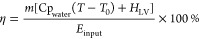
1where *m* is the evaporation rate (kg·m^–2^·h^–1^); *E*_input_ is the normal direct solar irradiation input (1000 W·m^–2^); Cp_water_ is the water specific heat capacity (4.2 kJ·kg^–1^·K^–1^); *T* and *T*_0_ are the surface average temperature and environment temperature respectively; *H*_LV_ is the water enthalpy of vaporization at the surface equilibrium average temperature (2434 kJ/kg at 28.2 °C, data from The Engineering ToolBox 2010 Water – Heat of Vaporization: https://www.engineeringtoolbox.com/water-properties-d_1573.html).

## Results and Discussion

### Suspending Evaporator Conception

**Figure 1:**
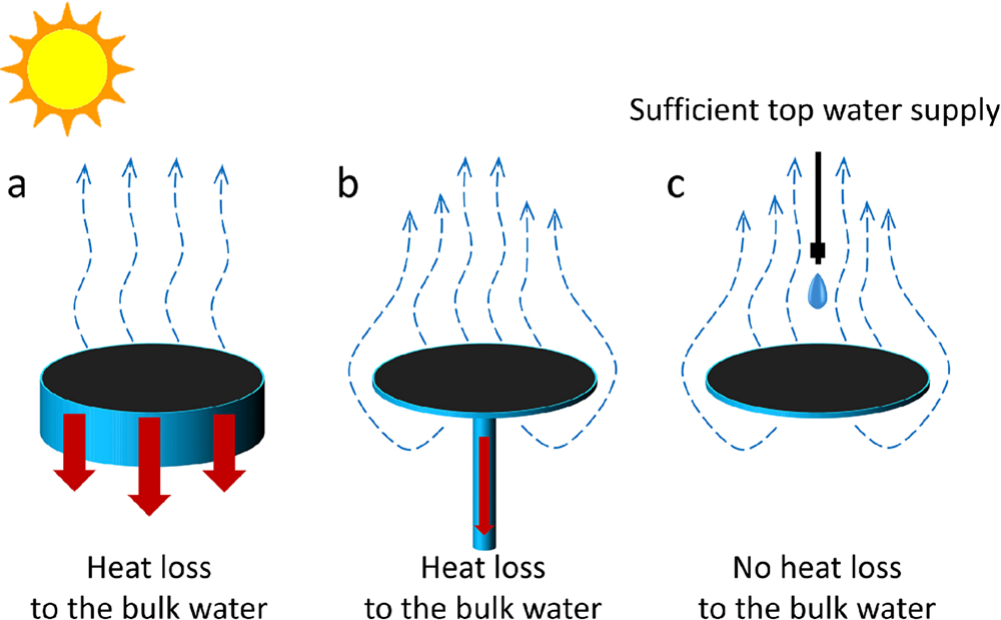
(a) Interfacial evaporator, (b) umbrella evaporator, and (c) suspending evaporator comparison in vapor transport conditions and heat loss.

At present, IEs are the most widely studied, which float at the water–air interface with evaporation on the top surface (Figure 1a). The whole structure is floated on the water surface by a low-density thermal insulation layer, which helps reduce the heat loss to the bulk water below. Recently, an umbrella evaporator has drawn more and more attention (Figure 1b). It utilizes a pillar to support the evaporation surface and supply water to the top surface by capillary force. This umbrella evaporator achieves double-sided evaporation, which has been proved more efficient than single-sided evaporation.^42^ The suspending evaporator proposed in this work adopts top water supply to achieve double-sided evaporation and completely cuts off the heat loss to the bulk water (Figure 1c). In addition, the water supply rate should meet the evaporation rate to keep the system working efficiently. The water supply of interfacial and umbrella evaporators heavily relies on the capillary force of the water transport system, which limits their scale-up. The proposed top water supply system is able to provide sufficient water for evaporation.

### Evaporation Performance

**Figure 2:**
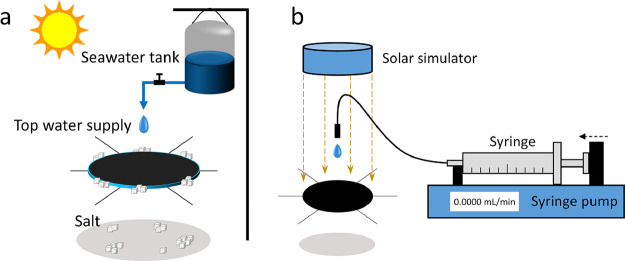
Schematic illustration of the application and experiment systems. (a) Schematic illustration of the suspending solar evaporator with the salt harvesting system. (b) Solar evaporation experiment system under 1 kW/m^2^.

**Figure 3:**
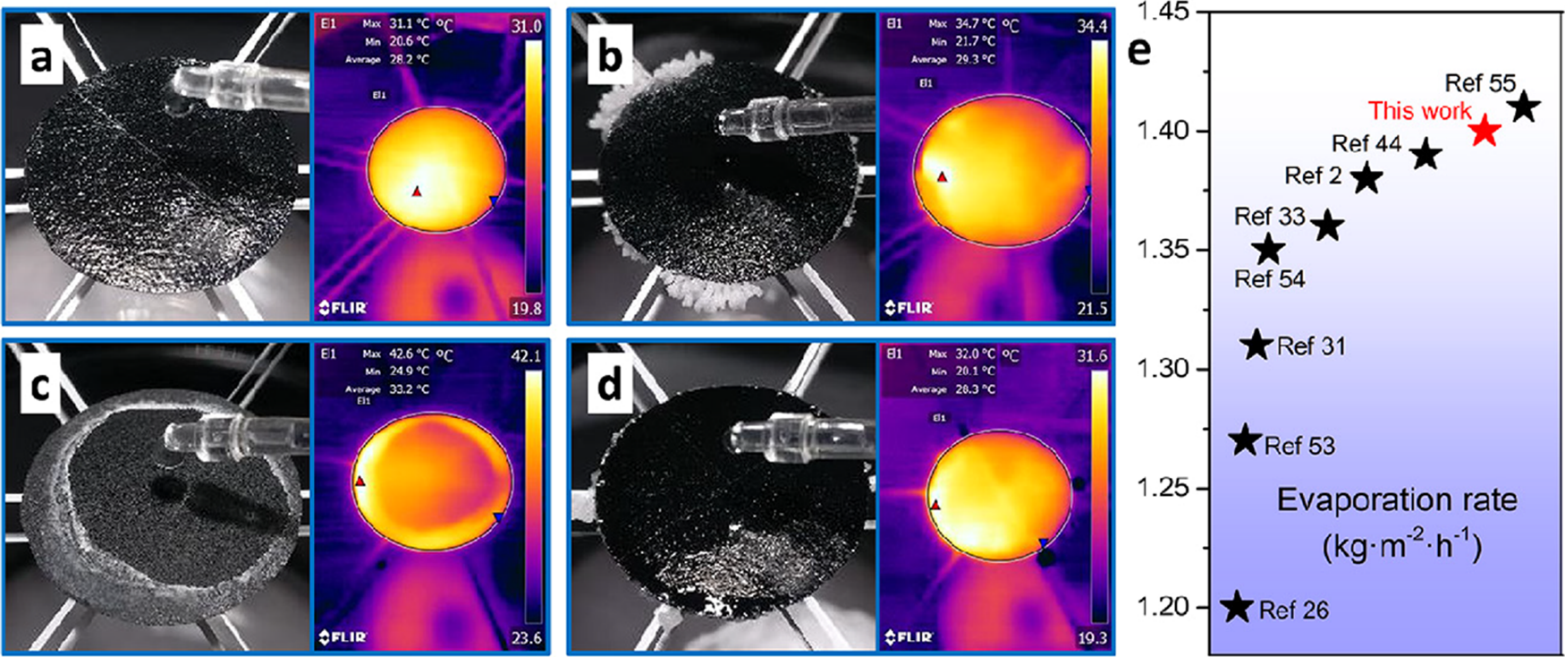
Solar evaporation performance of the suspending evaporator (3 cm in diameter) with top water supply. (a) Evaporation with DI water at 0.0165 mL/min. (b) Evaporation with 3.5 wt % NaCl solution at 0.0165 mL/min (100%). (c) Dry evaporation with 3.5 wt % NaCl solution at 0.0132 mL/min (80%). (d) Wet evaporation with 3.5 wt % NaCl solution at 0.0215 mL/min (130%). (e) Evaporation rate comparison between this work and previous reports under 1 sun.

Figure 2a shows the schematic view of the application of the suspending solar evaporator. Water was supplied from the top using a syringe pump that can control the flow rate (Figure 2b). The suspending evaporator (3 cm in diameter) works steadily with DI water at the top water supply rate of 0.0165 mL/min, corresponding to 1.40 kg·m^–2^·h^–1^, and the corresponding energy conversion efficiency is 95.7% (Figure 3a). The surface average temperature is 28.2 °C, which is much lower than that of a typical interfacial evaporator (40.3 °C) under the same test environment in our previous work.^44^ For this suspending evaporator, a lower surface average temperature indicates higher energy conversion efficiency. The energy loss for the suspending evaporator only comes from the heat loss to the environment, mainly due to natural convection and thermal radiation.^45^ Lower surface average temperature decreases the temperate difference with the surrounding environment, leading to a higher efficiency. At the same time, we also noted that several studies have reported that the evaporation enthalpy could be reduced in a porous evaporation media.^46−49^ This enthalpy reduction effect may also be involved in this work and needs to be further verified, while the accurate measurement of this enthalpy reduction remains challenging.^50,51^ The enthalpy reduction effect may also help explain this high apparent efficiency. It should be mentioned that the energy input for this evaporator mainly comes from the top solar irradiation without environmental energy attracted from the back surface.^52^ The reason is that the back surface temperature should be still higher than the environment because it is hard to maintain such a high temperature difference (7.2 °C) in such a thin evaporation surface (0.16 mm), which is saturated with water (80.6%). Notably, the evaporator can achieve salt harvesting from the evaporation surface edge at the same salt water supply rate (0.0165 mL/min) with a surface average temperature of 29.3 °C (Figure 3b). The suspending evaporator can work with a wide range of water supply. Lower water supply (0.0132 mL/min, i.e., 80% of the steady rate) makes the surface dry, and the salt accumulates to the center instead of at the edge (Figure 3c), while the system works well again when it is set back at the steady rate. This evaporation process with the surface becoming dry due to insufficient water supply is called dry evaporation. Insufficient water supply increases the surface average temperature to 33.2 °C, indicating a lower efficiency. By contrast, a higher water supply (0.0215 mL/min, i.e., 130% of the steady rate) makes the surface wet and salt-free. Excess water takes the salt down by drops, leaving few salt precipitates at the edge (Figure 3d). This evaporation process with water free flowing on the wet surface due to excess water supply is called wet evaporation. Excess water supply decreases the surface average temperature to 28.3 °C, which is almost equal to the DI water case. The evaporation rate comparison between this double-sided suspending evaporator and previous reports is shown in Figure 3e.^53−55^

### Simulation of Salt Distribution

To better understand the salt accumulation behaviors on the evaporation surface, we simulated the salt water flow and salt accumulation process using a two-dimensional plane model using the commercial software COMSOL Multiphysics. The salt transport is achieved by both fluid flow and diffusion in water. The fluid flow (Darcy’s law) and salt transport processes are used in the model. The fluid flow is a steady-state process, which is simulated first. The velocity magnitude results obtained are provided to the salt transport process for its dynamic process calculation. The evaporation surface was set as a porous media that can transport water by capillary force. The water supply was continuous from a center part circle (3 mm in diameter). The diameter of the evaporation surface was 3 cm, and its temperature was set uniform at 29.3 °C. For simplification, we set each part of the surface to evaporate at a uniform rate of 1.40 kg·m^–2^·h^–1^.

Darcy’s law is applied to simulate the water flow in the porous media, and the NaCl diffusion coefficient in water is modified by the Bruggeman model to fit in the porous media. Water properties derive from the software built-in database. For fluid flow,
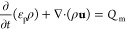
2
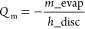
3
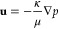
4where ε_p_ is the porosity of the porous media (0.806), ρ is the fluid density, and **u** is the fluid velocity. *Q*_m_ is the mass source which relates to the evaporation rate (*m*_evap: 1.40 kg·m^–2^·h^–1^), *h*_disc is the thickness of the evaporating layer (0.16 mm), κ is the permeability of the porous media (1 × 10^–13^ m^2^), μ is the fluid viscosity, and *p* is the fluid pressure. For salt diffusion:
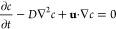
5
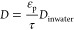
6where *c* is the NaCl concentration, *D* is the NaCl diffusion coefficient in the porous media, *D*_inwater_ is the NaCl diffusion coefficient in water (1.5 × 10^–9^ m^2^/s), and τ is the tortuosity of the porous media, which can be obtained by the Bruggeman model as follows:
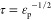
7

The pressure at the inlet boundary is set zero; thus, water can flow in when evaporation decreases the internal pressure. The flow rate is controlled by pressure to reach mass balance. The inlet concentration of salt water is 0.6 mol/L (containing 3.5 wt % NaCl) to simulate seawater. The initial NaCl concentration on the surface equals to the inlet concentration (0.6 mol/L), meaning that the surface is prewetted by salt water. The initial pressure is zero. An extremely fine mesh of the evaporation surface was automatically generated by COMSOL. The dependence of the mesh size has been tested, and its influence is negligible. The simulation results agree well with the experiment results, which can be directly validated by the salt distribution results in the following sections.

**Figure 4:**
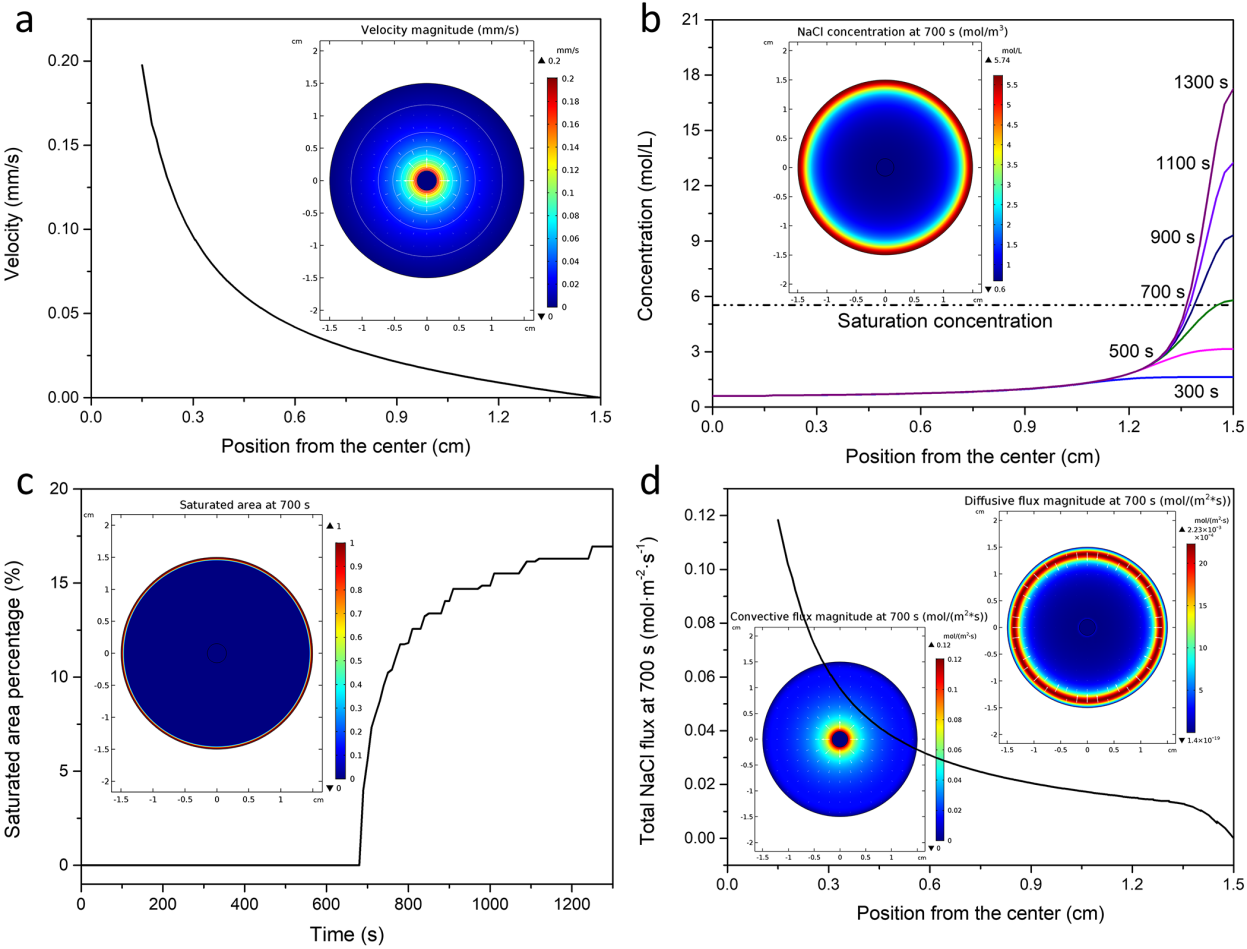
Simulation results of the salt distribution on the evaporation surface. (a) Velocity distribution along the radius. Inset is the velocity magnitude. (b) Salt concentration distribution along the radius from 300 to 1300 s. Inset is NaCl concentration magnitude at initial saturation time (700 s). (c) Saturated area percentage with time. Inset is the saturated area (red ring) at initial saturation time (700 s). (d) Total NaCl flux at initial saturation time (700 s). Insets are the convective and diffusive flux magnitudes at 700 s.

The salt water flows from the center to the edge accompanied by evaporation, and along the flow, the velocity rapidly decreases at first and then decreases steadily to the minimum at the edge, whose maximum velocity is 0.2063 mm/s (Figure 4a). The salt water flow carries NaCl to the edge and its concentration increases with evaporation. At 700 s, the edge reached saturation (27 wt % at 29.3 °C). Although salt accumulation will block the salt water flow, which affects salt transportation on the evaporation surface, the salt precipitation process takes time and tends to first occur on the surface instead of inside the channels.^56^ Hence, we calculated a little further (1300 s) to show the saturated area variation trend shortly after reaching saturation. With evaporation, the NaCl concentration increases rapidly only at the edge, leaving most parts of the surface unsaturated (Figure 4b). This edge accumulation behavior makes it possible to harvest salts from the edge and leaves the remaining surface work normally. After a certain salt accumulation time, the saturated area percentage soars from 0 in a short time and its growth slows down in the near following time (Figure 4c). Salt distribution on the evaporating surface is determined by both convective mass transfer to the edge and diffusive mass transfer to the center. At the beginning of saturation (700 s), the average convective flux is 0.0215 mol·m^–2^·s^–1^ and the average diffusive flux is 3.192 × 10^–4^ mol·m^–2^·s^–1^. The convective flux is over 67 times larger than the diffusive flux; thus, the convective flux dominates the salt distribution. Therefore, the total NaCl flux field is similar to the velocity field, with a slight difference at the edge due to back diffusive flux (Figure 4d).

### Water Distribution for Larger Area Evaporation

**Figure 5:**
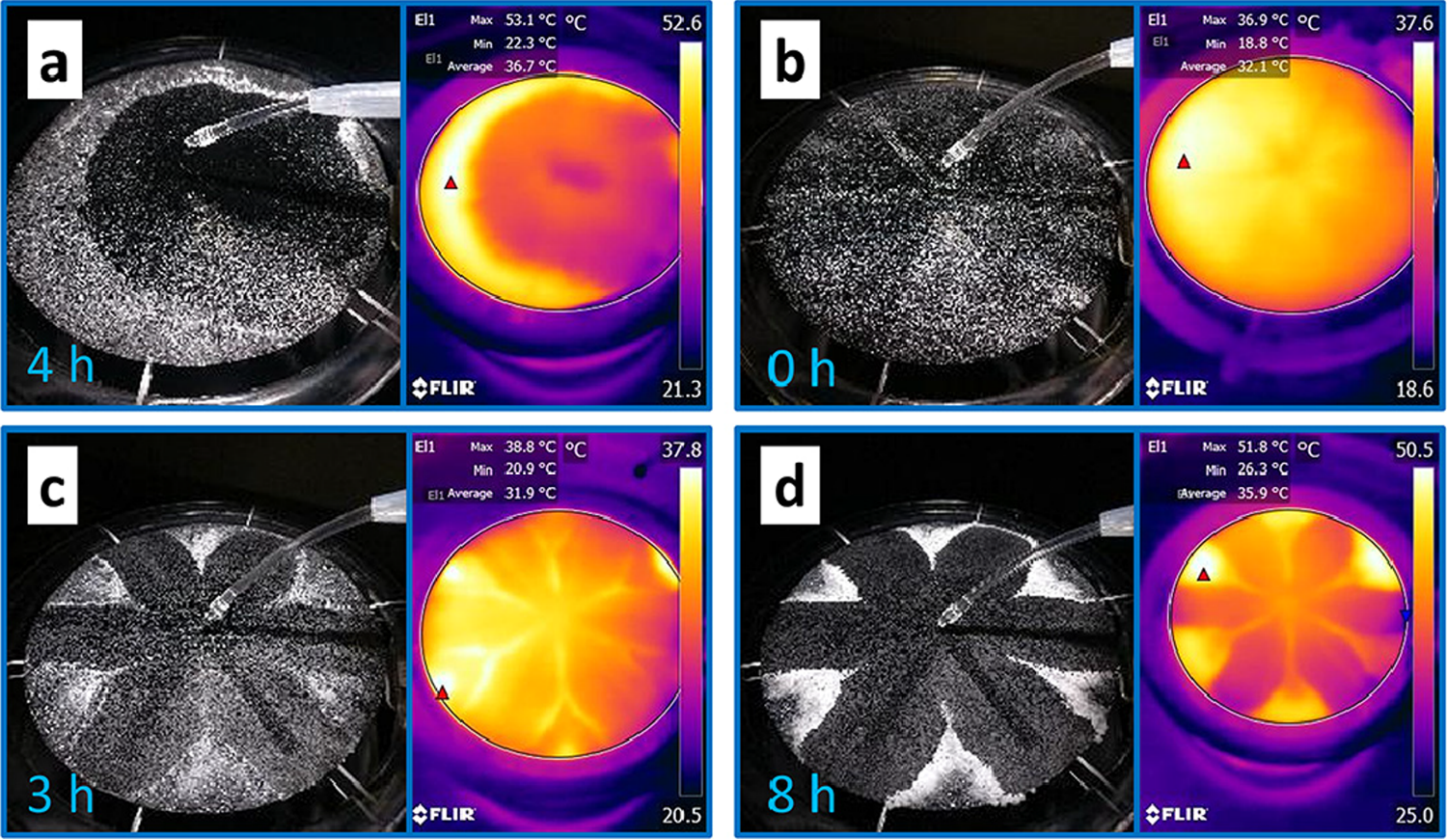
Salt water (3.5 wt % NaCl solution) evaporation and salt distribution on larger evaporation surfaces (11 cm in diameter). (a) Salt water evaporation. (b–d) Salt water evaporation and salt distribution with a radial arterial water distribution system at the salt water supply rate of 0.1650 mL/min (i.e., 1.04 kg·m^–2^·h^–1^) at 0, 3, and 8 h respectively.

In theory, if the salt water supply is sufficient, central salt water supply would accumulate the salt at the edge of the evaporation surface regardless of the surface diameter. However, it is not easy to uniformly distribute the salt water on the whole surface. A larger evaporation surface requires a faster water supply rate to keep up with the evaporation rate, for example, the average flow rate on the 3 cm surface is 0.0229 mm/s and that of the 11 cm case reaches 0.0937 mm/s from the simulation. It is not easy to transport salt water to the surface edge in time by capillary force. Increasing the top central water supply can only make the water flow to one side edge in a path of least resistance, leaving the other side covered by salt gradually (11 cm in diameter, Figure 5a). The average surface temperature reached 36.7 °C with the hottest point of 53.1 °C at 4 h. To solve the water nonuniform distribution and inefficient water transport to the edge, we designed a radial arterial water distribution system. It consists of 6 radial branches, which are made of airlaid paper and also coated by polypyrrole. Because of the additional better water transport of the airlaid paper and the water flow between the evaporation surface and the airlaid paper driven by capillary force, water first quickly runs to the edge through the branches and then wets all the remaining surface at the salt water supply rate of 0.1650 mL/min (i.e., 1.04 kg·m^–2^·h^–1^, Video S1, Figure 5b). The radial arterial water distribution system solves the water inefficient distribution problem, while it accumulates the salt on the surface in parts instead of at the edge (Figure 5c, d). Salt accumulated on the surface cannot fall down automatically for harvesting. At the same time, the surface average temperature also increased from 32.1 to 35.9 °C, and the hottest point increased more obviously from 36.9 to 51.8 °C, indicating that the efficiency is decreasing.

**Figure 6:**
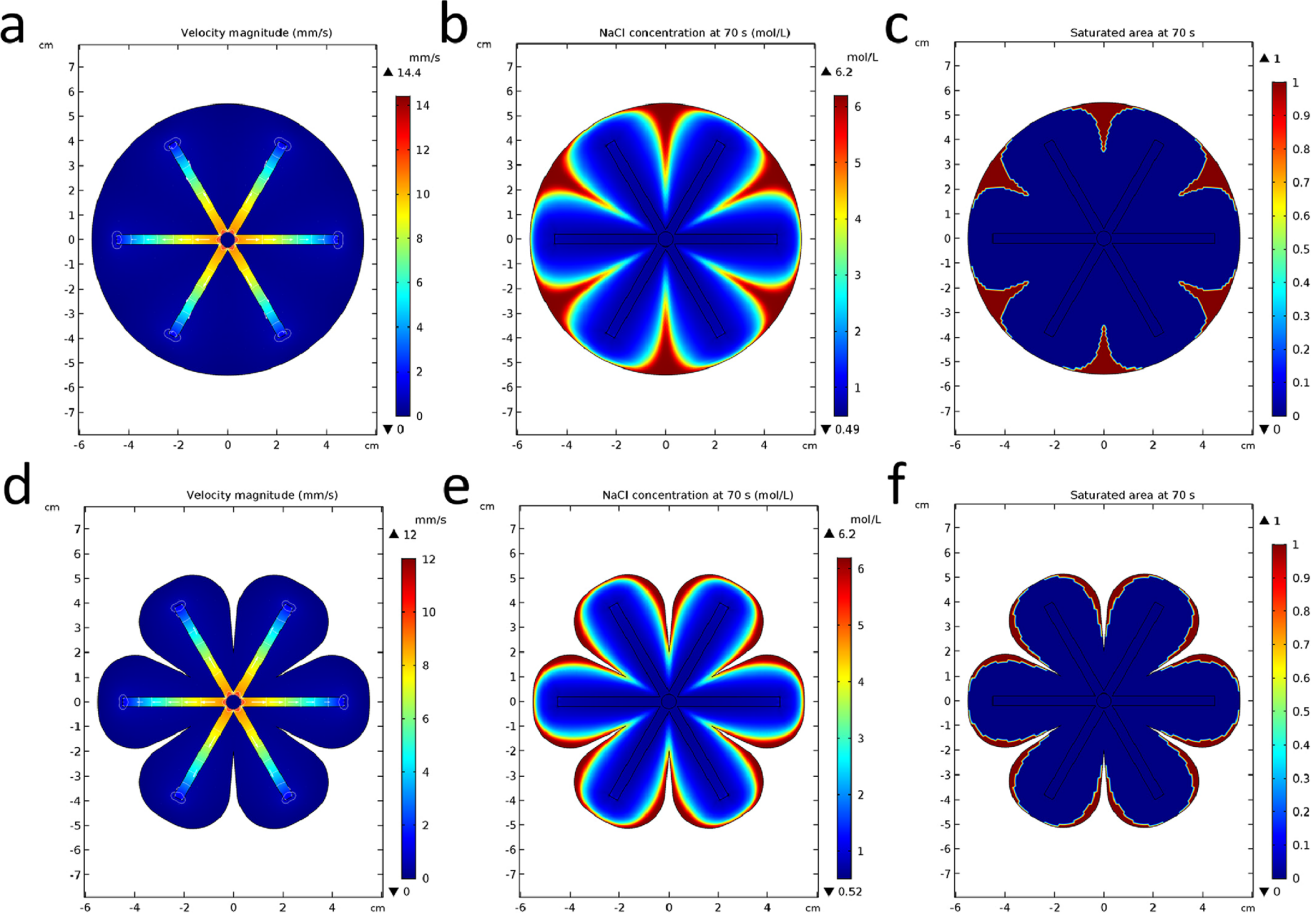
Simulations of salt distribution on the evaporation surfaces (11 cm in diameter) with the radical arterial water distribution system. (a) Velocity magnitude. (b) NaCl concentration at the initial saturation time (70 s). (c) Saturated area at the initial saturation time. (d) Velocity magnitude of the floriform surface. (e) NaCl concentration at the initial saturation time (70 s) of the floriform surface. (f) Saturated area at the initial saturation time of the floriform surface.

To solve the salt accumulation on the surface problem, we did simulations of the salt distribution on the evaporation surface with the radial arterial water distribution system. To ensure a sufficient water supply in the distribution system, we set the permeability (κ) of the distribution system to 100 times that of the evaporation surface. The water distribution system changed the original water supply path, and the water supply in the radial branches is obviously faster than the remaining surface (Figure 6a). The parts far from the center and the radial branches have the slowest water supply rate. It only takes 70 s to reach initial saturation for this larger evaporation surface (11 cm in diameter and 700 s for the 3 cm case above, Figure 6b). The saturated area is the same area with the slowest water supply rate (Figure 6c), and the simulation result agrees well with the experiment result in Figure 5d. To make the salt accumulate at the edge for automatic falling, we cut out the saturated area (16.4%) to create a floriform evaporation surface (Figure 6d). The salt only accumulates at the edge, leaving the main surface at a low concentration for evaporation (Figure 6e). The saturated area at the edge makes it possible for the accumulated salt to fall down (Figure 6f).

**Figure 7:**
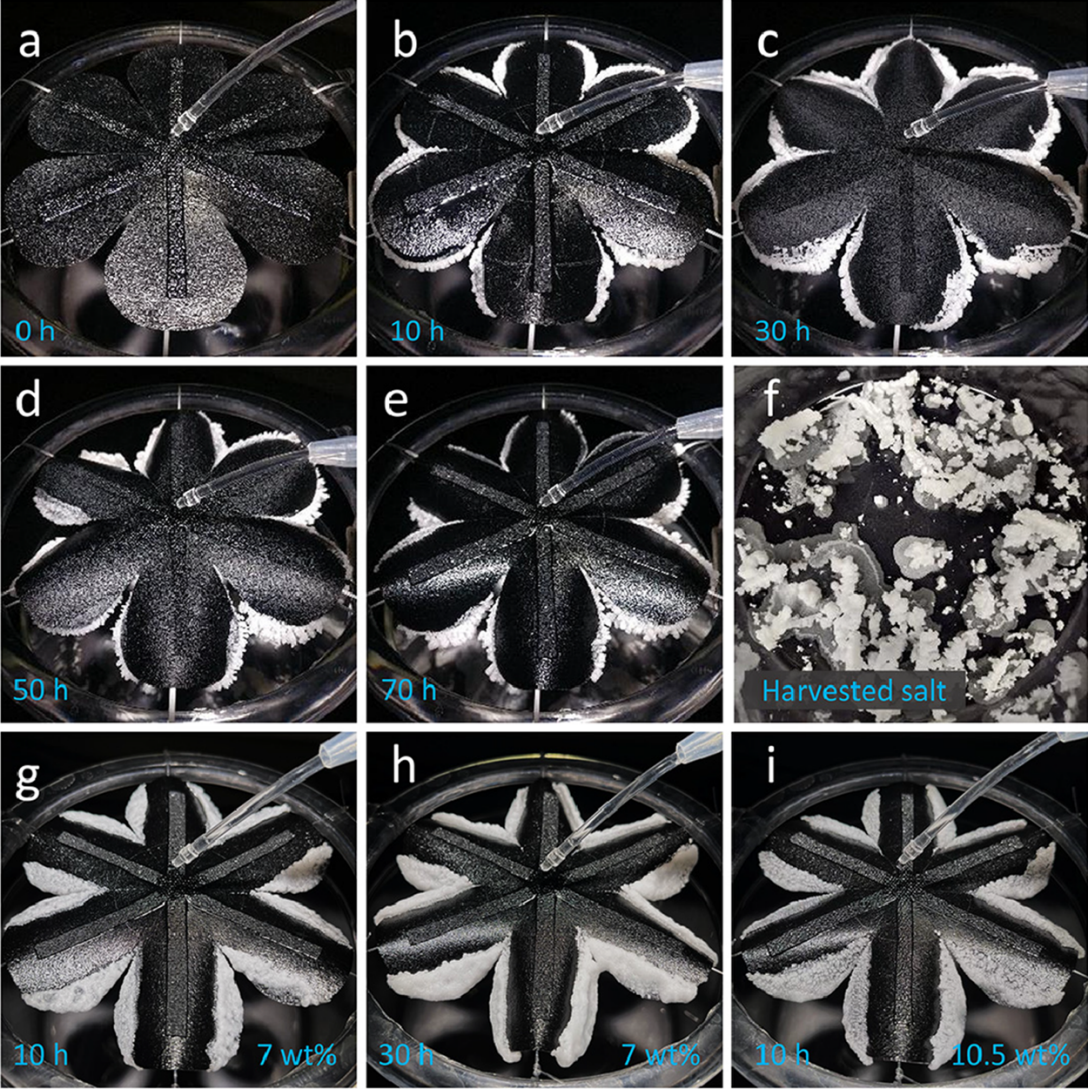
Salt water evaporation and salt harvesting from the floriform evaporation surface (11 cm in diameter). (a–e) Salt water evaporation with 3.5 wt % NaCl solution at 0.1650 mL/min with salt harvesting at 0, 10, 30, 50, and 70 h, respectively. (f) Harvested salt at 70 h. (g, h) Salt water evaporation with 7 wt % NaCl solution at 10 and 30 h. (i) Salt water evaporation with 10.5 wt % NaCl solution at 10 h.

Based on the simulation, we developed a floriform evaporator and conducted the salt water (3.5 wt % NaCl solution) evaporation experiments (11 cm in diameter). The experiment results agree well with the simulation results (Figure 7a–e). Salts only accumulate at the edge and fall automatically for harvesting. The evaporation continuously ran for 70 h at the evaporation rate of 0.1650 mL/min (i.e., 1.04 kg·m^–2^·h^–1^ based on the complete circle area and 1.24 kg·m^–2^·h^–1^ based on the actual surface area), which is the same as the uncut surface. This is due to both the robustness of the evaporator and the influence of the salt accumulation on the uncut surface. The harvested salts in 70 h are shown in Figure 7f. We further tested its evaporation performance with higher NaCl concentrations, that is, 7 wt % and 10.5 wt % NaCl solution (2 and 3 times the seawater salt content). For 7 wt % NaCl solution, salt covers a large area around the edge and grows thicker with time without salt falling (Figure 7g, h). The scenario of 10.5 wt % NaCl solution is similar, while the salt grows faster (Figure 7i). Higher salt concentration leads to an earlier saturation before reaching the edge, which makes the salt cover the surface around the edge without salt harvesting.

## Conclusions

Here, we demonstrated a double-sided suspending evaporator with top water supply and surface water distributor for high-efficient concurrent solar evaporation and salt harvesting for large area applications. Both sides of this evaporator can evaporate water with automatic salt harvesting from the edge. Top water can supply sufficient water to the evaporation surface for large area applications and cut off the heat loss to the bulk water. The energy conversion efficiency (3 cm in diameter case) reached 95.7% at 1.40 kg·m^–2^·h^–1^ with deionized water under 1 sun with a remarkable low surface average temperature (28.2 °C). By both simulations and experiments, a radial arterial water distribution system on the evaporation surface was designed to efficiently distribute water on a larger evaporation surface (11 cm in diameter). The water transport path was altered using the water distribution system, which leads to salt accumulation on the surface body, where salt cannot be harvested. By cutting out the salt accumulation area (16.4%), a floriform evaporator was obtained, which forcedly exposes the salt at the edge for harvesting. We achieved 70 h continuous solar evaporation and salt harvesting at 1.04 kg·m^–2^·h^–1^ (based on the complete circle area with 11 cm in diameter and 1.24 kg·m^–2^·h^–1^ based on the actual surface area) on the floriform evaporation surface. This work resolves the scale-up problems of the solar evaporators and advances new ideas for the structural design of the evaporators for high-efficient large area applications.

## Supporting Information Available

Water distribution and salt accumulation on the floriform evaporator (MP4)
